# Infant feeding by South African mothers living with HIV: implications for future training of health care workers and the need for consistent counseling

**DOI:** 10.1186/s13006-019-0205-1

**Published:** 2019-02-14

**Authors:** Nora S. West, Sheree R. Schwartz, Nompumelelo Yende, Sarah J. Schwartz, Lauren Parmley, Mary Beth Gadarowski, Lillian Mutunga, Jean Bassett, Annelies Van Rie

**Affiliations:** 1 Johns Hopkins Bloomberg School of Public Health, 615 N Wolfe Street, Baltimore, MD 21205 USA; 2 Witkoppen Health and Welfare Centre, 105 William Nicol Drive, Fourways, Johannesburg 2055 South Africa; 3 University of North Carolina School of Public Health, 135 Dauer Drive, Chapel Hill, NC 27599 USA; 4 University of Antwerp, Prinsstraat 13, 2000 Antwerpen, Belgium

**Keywords:** Infant feeding, HIV, Counselling

## Abstract

**Background:**

Since 2010, the World Health Organization recommends lifelong antiretroviral treatment for all women living with HIV, and exclusive breastfeeding for six-months followed by breastfeeding until 24-months for all HIV positive mothers. Nevertheless, many mothers living with HIV do not initiate breastfeeding or stop prematurely, and many countries are still in the process of updating their national infant feeding guidelines to align with World Health Organization recommendations. We sought to understand uptake of breastfeeding and factors that influence decision-making regarding infant feeding in women living with and without HIV who receive ante- and postnatal care at a primary healthcare setting.

**Methods:**

Programmatic data on infant feeding intentions and practices among women attending an ante-and postnatal clinic service at a primary care clinic in Johannesburg, South Africa were summarized using descriptive statistics. Qualitative interviews were conducted with 12 healthcare providers, 12 women living with HIV who were breastfeeding and 10 who were formula feeding. Interviews were analyzed using a content analysis approach.

**Results:**

Pregnant women living with HIV were less likely to express an intent to breastfeed (71% vs 99%). During the first 6 months postpartum, mothers living with HIV were also less likely to exclusively breastfeed compared to HIV-negative mothers. Mixed messages during infant feeding counselling, social and economic factors, and fear of HIV transmission influenced women’s choices to initiate and continue breastfeeding.

**Conclusions:**

As infant feeding guidelines for women living with HIV have evolved rapidly in the past 10 years, uniform messages on the low risk of mother-to-child transmission of HIV associated with breastfeeding while on ART and on introduction of complementary foods after 6 months of age are needed.

## Background

Since 2010 the World Health Organization (WHO) has recommended that women living with HIV be counselled to exclusively breastfeed for the first 6 months of the infant’s life, with introduction of complementary foods after 6 months and continued breastfeeding up to 24 months [1]. In the Option B+ and ‘Treat All’ era, where pregnant women living with HIV are initiated on lifelong antiretroviral therapy (ART) independent of their CD4 count or co-morbidities, HIV transmission risk from mother to child has been reduced to less than 2% among mothers adherent to treatment [2, 3]. Alongside the rapidly evolving HIV treatment guidelines and global ART scale-up, guidelines for infant feeding among women living with HIV in South Africa have changed substantially over the past 15 years, from government-supplied formula feeding in 2002 and cessation of breastfeeding at 6 months in 2011, to exclusive breastfeeding for 6 months and continued breastfeeding up to 24 months for all women on ART in 2017 [4].

Breastfeeding provides infants with important nutrients and antibodies, and is associated with lower risk of malnutrition and diarrheal illnesses, which are among the leading causes of infant mortality in low and middle income countries [1]. Women living with HIV face important barriers to breastfeeding, including fear of HIV transmission and inadvertent HIV status disclosure [5]. In sub-Saharan Africa (SSA), the predominant type of breastfeeding for infants less than 6 months of age is mixed feeding, which is defined as breastfeeding supplemented with liquid or solid food alongside breast milk prior to 6 months of age [6, 7]. Because of its contribution to increased risk of mother-to-child transmission of HIV, mixed feeding for women living with HIV has been discouraged in the South African infant feeding guidelines since 2007 [8, 9].

Healthcare workers’ views and opinions may influence uptake, continuation, and cessation of breastfeeding among women living with HIV [5]. Furthermore, societal, familial, occupational and economic factors can also impact a mother’s infant feeding choices [5]. The purpose of this study was to document the uptake of breastfeeding among women in a primary healthcare setting and conduct an in-depth qualitative exploration to understand how guidelines, infant feeding counseling, and individual, familial and societal factors influence infant feeding choices. We considered perspectives of both healthcare providers and women living with HIV, in order to more comprehensively identify facilitators and barriers to uptake of current infant feeding recommendations.

## Methods

### Setting

Study participants were enrolled at Witkoppen Health and Welfare Centre (Witkoppen), a large primary care clinic located in Johannesburg, South Africa. Witkoppen is a nongovernmental organization that operates in partnership with the South African Department of Health (DOH). Since 2014, the clinic implemented a ‘FRESH Start’ program where women receive integrated HIV and antenatal care (ANC) during pregnancy, and integrated HIV and postnatal care (PNC) for mother-infant dyads up until 18 months postpartum. HIV care included HIV testing at first antenatal care visit and immediate initiation of ART for all women living with HIV. HIV negative women were retested for HIV every 3 months during pregnancy and the postpartum period. In the postnatal period women were encouraged to attend the clinic with their babies at 7 days, 6 weeks, 10 weeks, 14 weeks, 6 months, 9 months, 12 months and 18 months. Infants born to women living with HIV were tested for HIV at 6 weeks of age.

At the time of qualitative data collection, Witkoppen service delivery followed the 2013 DOH Infant and Young Child Feeding Policy which recommended exclusive breastfeeding for 6 months, and continued breastfeeding for up to 12 months alongside complementary foods for women on ART [9]. Witkoppen service delivery was updated in-line with the amended feeding policy in July 2017, which recommends continued breastfeeding for up to 24 months. All women attending Witkoppen received infant feeding information from a health educator in group format while waiting to see a nurse-clinician, individual infant feeding counselling from lay counsellors, counselling by a dietician if feeding challenges were recorded. Nurse clinicians further reinforce messages about infant feeding during one-on-one clinical visits.

### Programmatic data

HIV status and data on infant feeding practices routinely collected at all visits were extracted from the Witkoppen’s electronic FRESH start database. The FRESH start database documents all women’s planned method of infant feeding during the ANC period, and current infant feeding method at each PNC visit. The intent and uptake of exclusive breastfeeding was described and compared between HIV positive and negative women. Analyses were conducted using STATA 14.1 (College Station, Texas, USA).

### Qualitative data

We conducted 34 semi-structured in-depth interviews (IDIs) between July and August 2015: 22 with mothers (age ≥ 18 years) living with HIV on ART who had a child less than 12 months of age and who were breastfeeding (*n* = 12) or formula feeding (*n* = 10), and 12 with healthcare providers engaged in ante-and postnatal care. Healthcare providers were selected to represent a diversity of roles, including nurses (*n* = 4), lay counselors (*n* = 6) and health educators (*n* = 2). All participants were recruited purposively by study staff and with the help of Witkoppen personnel.

IDIs were performed in English or Zulu and audio recorded. Interviews were conducted by female interviewers trained in qualitative interviewing. Using a topical interview guide, IDIs explored the following domains of interest: influences on feeding method choices, understanding of infant feeding choices, experiences with feeding decision-making for HIV-positive mothers, clinic-based messaging around feeding options for HIV-positive women, understanding of the current breastfeeding guidelines for HIV-positive mothers and implementation of the guidelines. Summary notes were completed by the interviewer immediately following the interviews. Interviews were transcribed and translated into English as necessary.

The study used a content analysis approach [10], with coding structured around pre-defined and latent content. Through an iterative process of reviewing the transcribed data, coders developed and discussed codes, compared codes for accuracy and interpretation, and finalized a codebook. IDI transcripts were then coded by two independent coders (N.W. coded all transcripts with N.Y, L.P., and M.B.G. sharing coding responsibility as second coders). Coded transcripts were compared for accuracy and interpretation, and discrepancies discussed and resolved. Themes produced from the coding were reviewed and discussed, and illustrative quotes selected to represent the themes.

## Results

### Intent to and uptake of exclusive breastfeeding during the first six months postpartum

**Fig. 1:**
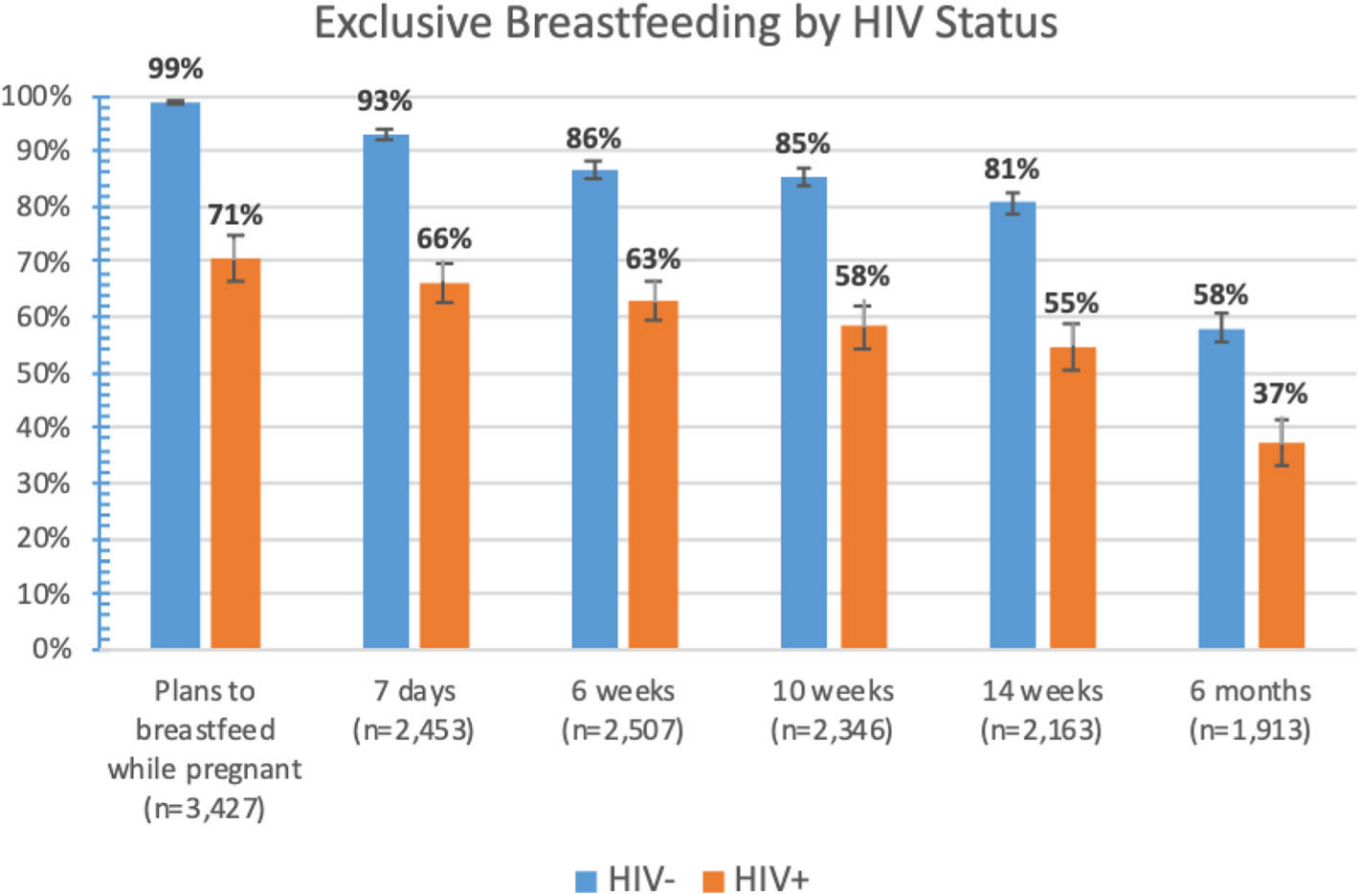
Exclusive breastfeeding intent and practices among women receiving care at a primary care clinic in Johannesburg between July 2015 and March 2018, stratified by HIV status and timing of visit

Among 8116 women attending antenatal or postnatal care at Witkoppen between July 7, 2015 and March 6, 2018, 1613 (19.9%) were HIV-positive. Most pregnant women expressed an intent to breastfeed (95.1, 95% CI: 94.3, 95.8), but women not living with HIV were more likely to plan to breastfeed compared to women living with HIV (98.9% vs 70.6%, *p* < 0.001) (Fig. 1). At the first postnatal visit, most women self-reported having initiated breastfeeding (86.3, 95% CI: 84.9, 87.6), but women living with HIV were again less likely to have initiated breastfeeding compared to women not living with HIV (93.1% vs 66.3%, p < 0.001). The proportion of mothers self-reporting exclusive breastfeeding decreased over time, from 86.3% at day 7 to 73.1% at week 14 and 51.7% at month six. At all time points, the proportion of women reporting exclusive breastfeeding was higher among women free of HIV compared to those living with HIV.

### Barriers and facilitators of exclusive breastfeeding among mothers living with HIV

The 22 women living with HIV participating in an IDI (median age 31 years, median age of their babies 16 weeks) described a constellation of individual, economic, structural, and social factors that influenced feeding choice and breastfeeding practice.

### Fear of vertical HIV transmission

Even though healthcare providers counselled their clients on the benefits of breastfeeding and discussed risks so women could weigh HIV transmission risk to the benefits of breastfeeding, for women who chose to formula feed, any element of HIV transmission risk outweighed the benefits of breastfeeding:

“They are being counselled to breastfeed, but also warned about the potential dangers of mixed feeding, [HIV] transmission, and because some of the mixed feeding may be out of their control, out of an abundance of caution they choose formula feeding.” -HIV Counsellor

Most women expressed a fear of transmission of HIV to their infants. Among all women who had opted for formula feeding, the decision to do so was driven by fear of HIV transmission to the baby:

“I didn’t want any chance for them [the infant] to get HIV. I felt that they [healthcare providers] said if you are positive and take your medication properly, then you can have a negative baby. I decided that I don’t want any chance.” –Woman living with HIV, age 37, formula feeding

Even women who breastfed uniformly expressed concerns around transmission of HIV to their baby, and how this generated uncertainty around their feeding choice:

“As you know your status you would want to breastfeed but sometimes you would think that if your baby would end up being like that, if he becomes positive I will always blame myself. You will do whatever you can but you will not be happy, you will just breastfeed.” –Woman living with HIV, age 32, breastfeeding

Some women mentioned that a lack of peer models or tangible examples demonstrating that feeding was safe had influenced their decision-making:

“If I can talk to someone who is HIV positive who has breastfed the baby and the baby is HIV negative then I’ll do it [breastfeed], because so far I have only talked to the ladies who bottle fed their babies and they [the babies] are negative. I think that’s what influenced me to make the decision of bottle feeding him; I’ve never talked to someone who has breastfed and the baby is negative.” –Woman living with HIV, age 24, formula feeding

### Inconsistent messaging delivered by healthcare providers

Although providers overall were supportive of breastfeeding over formula feeding when mothers were virally suppressed, many providers acknowledged that women living with HIV may receive inconsistent messages related to infant feeding:

“I think breastfeeding is like falling pregnant, there are health care workers who would encourage an HIV positive woman to fall pregnant and there are those who won’t encourage HIV mothers to breastfeed because they are afraid that they will infect their babies.” –Nurse

Many providers also expressed that inconsistent messages regarding infant feeding for mothers living with HIV could be the result of frequently changing guidelines:

“If maybe we could sing the same song and all that, then people will be having the same information about that [infant feeding]. The disadvantage, again is that then government will change it [infant feeding guidelines].” –Lay Counsellor

### Confusion around exclusive breastfeeding and continued breastfeeding after six months postpartum

The majority of mothers living with HIV expressed concerns and confusion around continued breastfeeding after the introduction of complementary foods at 6 months. The overall perception was that, contrary to the guidelines, breastfeeding must stop at 6 months with the introduction of any other foods:

“They told us that when the baby is six months you can start feeding him and you can then stop breastfeeding. Before you start feeding him at 6 months you have to stop breastfeeding and he starts with food.” -Woman living with HIV, age 31, breastfeeding

Confusion over the transition from exclusive breastfeeding was also pervasive among health care providers:

“After how many years since we stopped giving formula, I see they [providers] wrote in the file ‘stop breastfeeding at six months’ and there’s no reason why they should stop. The viral load is undetectable. There’s really no reason. And the moms come and cry here and they don’t have money for milk. So, I think that’s our biggest thing and if we get that right, I think the rest will just start to follow, getting more people to breastfeed.” -Health Educator

Tied to the confusion about the guidelines for feeding after 6 months was a pervasive misunderstanding about when mixed feeding (combining breastfeeding with other liquids or solids) puts the infant at risk and should be discouraged (first 6 months) or when it should be encouraged (after 6 months):

“After 6 months they [the mother] can stop [breastfeeding] the child, the breastmilk. She can give the child the formula and the food cause the child, if she’s 6 month I think, she’s old, can give her anything. As long as [the baby] stopped breastfeeding.” –Lay Counsellor

### Social and economic influences

Many women living with HIV described social pressure from family and community that impacted their infant feeding decision. Some mothers expressed they had to create stories for family members to justify why they were not breastfeeding:

“She was very angry why I was not breastfeeding the baby, and I said ‘no the breast doesn’t pump,’ because I didn’t want to tell her. I don’t have a good relationship with my mother, so disclosing to her she’ll be very angry. So I said to her ‘my milk doesn’t come and the baby is already here at home, so there is nothing I can do; I have to bottle feed the baby’. And then after 4 to 5 days when I woke up she saw my breasts full with milk and she said ‘but you said there is no milk’ and I said ‘maybe it started today. And at the clinic they said if you are breastfeeding you must breastfeed from the first day and if you are bottle feeding you must bottle feed’, so I said I am going to bottle feed all the way, but she was very angry.” –Woman living with HIV, age 27, formula feeding

Many mothers who chose to breastfeed cited the cost of formula as a driver for their decision:

“I chose to breastfeed because I am not working and sometimes you may find that I would not have money to buy formula as the father also doesn’t have a good job.” -Woman living with HIV, age 31, breastfeeding

Working mothers highlighted that employment influenced their feeding choice. Concerns that family members or daycare providers caring for their child would feed them something other than breastmilk led some women to choose formula feeding over breastfeeding:

“After I have delivered my baby they [healthcare providers] told me to breastfeed and I didn’t take that advice because I knew I was going to work very soon. They told me that I could pump my breasts and freeze the milk, but it wasn’t ok because I knew my baby was going to crèche and they might give him water when he cries or food.” -Woman living with HIV, age 24, formula feeding

## Discussion

In this study we observed a large disparity in breastfeeding practices among HIV positive and negative women, with pregnant women living with HIV being significantly less likely to express an intent to breastfeed, mothers living with HIV being less likely to breastfeed at any point in time, and few women breastfeeding for more than 6 months. Infant feeding decisions were driven by a combination of fear of vertical HIV transmission, employment, financial constraints, social pressure, and information received from health care providers. Providers also expressed frustration with frequently changing guidelines. Confusion and inconsistent messaging regarding the benefits of continued breastfeeding after six-months was particularly high.

### Influences on decision-making and inconsistent counselling

Other studies have also reported low breastfeeding uptake and high early cessation of breastfeeding in South Africa [11, 12]. With regards to the factors influencing infant feeding choices in the context of HIV-infection, many of our findings mirror key themes found in a metasynthesis of infant feeding attitudes and practices within the context of HIV from 16 qualitative studies across 13 countries in sub-Saharan Africa [5]. In this metasynthesis, fear of HIV transmission, family, cost and healthcare provider messages were all highly influential on infant feeding choice [5]. Infant feeding choice is thus multifactorial. Efforts to successfully implement new guidelines must therefore go beyond training providers on the changes in the guidelines.

In addition, we found that inconsistent messages during infant counseling was prevalent, with pregnant women and mothers not receiving counselling that is in-line with the most recent guidelines around feeding options. Similar observations were made in other studies in sub-Saharan Africa [13–17]. Due to the frequent changes in guidelines, formal training on infant feeding guidelines received may vary between providers. Consequently, the feeding cessation messages given may reflect the guidelines providers were formally trained in rather than the most recent guidelines. Some providers were aware of this and expressed frustration with frequently changing guidelines and were concerned about how this leads to the inconsistent infant feeding messages women receive.

A recent study from South Africa found that providers were likely to overemphasize the risk of HIV transmission in the postnatal period for women living with HIV [18]. We found confusion was particularly high regarding breastfeeding after 6 months postpartum. Many women and providers believed that a mother living with HIV should stop breastfeeding and switch to formula or other foods at 6 months postpartum, suggesting that a lack of clarity on guidelines among providers may be a salient factor. A study from Uganda also found that confusion about the initiation of mixed feeding after six-months impacted infant feeding decision making [17]. For many years, messages about the danger of mixed feeding during the first 6 months of an infant’s life have been very prominent in South African and other sub-Saharan African healthcare settings [9, 19].

The current South African guidelines emphasize that it is a mother’s right to make her own choice about infant feeding when provided all appropriate information [9]. This assumes mothers will receive all information in a clear and effective way within the healthcare system. Because ambiguous choice-focused counselling around infant feeding may result in confusion, clear and uniform recommendation-based counselling may be more effective [15]. Indeed, a study conducted in KwaZulu-Natal, South Africa found that structured counselling visits were strongly associated with adherence to exclusive breastfeeding [20]. In addition to adopting evidence-based counselling models that have already shown efficacy for breastfeeding among women living with HIV, community-based peer support models may also be beneficial [21]. In our study, a number of participants highlighted a lack of peer examples of women living with HIV who breastfeed and have babies that remain HIV-negative. Finally, community-level promotion of exclusive breastfeeding for all women, not only women living with HIV, is a critical component of reducing stigma and increasing adherence to the guidelines [20].

### Implications

Recognition of the complex infant feeding challenges is particularly timely as it has implications for the fidelity to and success of South Africa’s infant feeding policy. In July 2017, the South African DOH again changed their guidelines to align with WHO guidelines which recommend exclusive breastfeeding for six-months with continued breastfeeding for up to 24 months for women living with HIV on ART [4]. However, early cessation of breastfeeding among women living with HIV is likely to persist if the fear of any risk of HIV transmission to the infant, no matter how small is not recognized by the healthcare workers, and if the multiple and complex socio-economic issues influencing infant feeding choices are not adequately addressed. Furthermore, given the frustration expressed by providers about frequently changing guidelines, securing understanding and buy-in through continued engagement of providers is critical whenever new guidelines are introduced.

### Limitations

Our ability to obtain insights on the diverse factors that impact feeding decision-making at different time-points was strengthened by combining quantitative programmatic data collected at different time points with qualitative interview data from both healthcare providers and women living with HIV with infants of different ages. This study is however not without limitations. First, while the data allowed us to accurately determine if a woman was exclusively breastfeeding, once other foods were introduced it was not clear whether she was breastfeeding or using formula/milk in combination with solids. Second, our qualitative data are cross-sectional and do not capture infant feeding decision-making and its determinants over time.

## Conclusion

There is strong evidence that women living with HIV continue to be less likely to breastfeed their children despite the recommendation that all women living with HIV on ART breastfeed. The choice to initiate formula or breastfeeding, as well as the decision to continue breastfeeding beyond 6 months is a complicated process influenced by the healthcare provider counseling, fear for HIV transmission, individual economic realities, social pressure, and stigma. As countries in SSA adopt and rollout updates to infant feeding guidelines, women living with HIV are entitled to receive uniform and unambiguous messages that address the multi-level factors that influence their feeding choice and practice.

## Abbreviations

ANC: Antenatal Care
ART: Antiretroviral Therapy
DOH: Department of Health
IDI: In-depth Interview
PNC: Postnatal Care
SSA: Sub-Saharan Africa
WHO: World Health Organization
Witkoppen: Witkoppen Health and Welfare Centre
